# Dendritic cell-derived interleukin-15 is crucial for therapeutic cancer vaccine potency

**DOI:** 10.4161/21624011.2014.959321

**Published:** 2014-12-15

**Authors:** Yi Zhang, Shenghe Tian, Zuqiang Liu, Jiying Zhang, Meili Zhang, Marcus W Bosenberg, Ross M Kedl, Thomas A Waldmann, Walter J Storkus, Louis D Falo, Zhaoyang You

**Affiliations:** 1 Department of Dermatology; University of Pittsburgh School of Medicine; Pittsburgh, PA USA; 2 Lymphoid Malignancies Branch; Center for Cancer Research, National Cancer Institute; Bethesda, MD USA; 3 Departments of Dermatology and Pathology; Yale University School of Medicine; New Haven, CT USA; 4 Department of Immunology and Microbiology; University of Colorado; Aurora, CO USA; 5 Department of Immunology; University of Pittsburgh School of Medicine; Pittsburgh, PA USA; 6 University of Pittsburgh Cancer Institute; Pittsburgh, PA USA

**Keywords:** cancer vaccines, DC, *IL-15*

## Abstract

*IL-15* supports improved antitumor immunity. How to best incorporate *IL-15* into vaccine formulations for superior cancer immunotherapy remains a challenge. DC-derived IL-15 (DCIL-15) notably has the capacity to activate DC, to substitute for CD4^+^ Th and to potentiate vaccine efficacy making IL-15-based therapies attractive treatment options. We observed in transplantable melanoma, glioma and metastatic breast carcinoma models that DCIL-15-based DNA vaccines in which DC specifically express *IL-15* and simultaneously produce tumor *Aghsp70* were able to mediate potent therapeutic efficacy that required both host Batf3^+^ DC and CD8^+^ T cells. In an inducible Braf^V600E^/Pten-driven murine melanoma model, DCIL-15 (not rIL-15)-based DNA vaccines elicited durable therapeutic CD8^+^ T cell-dependent antitumor immunity. DCIL-15 was found to be superior to rIL-15 in “licensing” both mouse and human DC, and for activating CD8^+^ T cells. Such activation occurred even in the presence of Treg, without a need for CD4^+^ Th, but was IL-15/IL-15Rα-dependent. A single low-dose of DCIL-15 (not r*IL-15*)-based DC vaccines induced therapeutic antitumor immunity. CD14^+^ DC emigrating from human skin explants genetically-immunized by *IL-15* and *Aghsp70* were more effective than similar DC emigrating from the explants genetically-immunized by *Aghsp70* in the presence of rIL-15 in expressing membrane-bound IL-15/IL-15Rα and activating CD8^+^ T cells. These results support future clinical use of DCIL-15 as a therapeutic agent in battling cancer.

## Introduction

Generating robust, durable and effective tumor antigen (Ag)-specific CD8^+^ T cells that are competent to eradicate primary tumors and tumor metastases or to prevent disease recurrence as a consequence of active specific vaccines has proven clinically difficult.^1^ This may relate to significant hurdles including the inherently poor immunogenic nature of many tumors and tumor-induced immune suppression mediated by myeloid-derived suppressor cells (MDSC) and regulatory T cells (Treg).^1-3^ Deficiency of CD4^+^ T helper (CD4^+^ Th) cell numbers and/or functionality, which are usually needed to optimize CD8^+^ T cell responses,^4^ has further dampened hope for the generalized effectiveness of conventional vaccine strategies in the vast majority of cancer patients.

Dendritic cells (DC), key players in the host handling of injected vaccines (e.g., professionally processing and presenting vaccine Ag and functionally polarizing cognate Ag-specific T cells), are crucial for activating potent Ag-specific T cell responses.^1^ *In vitro*-generated DC or *in vivo* DC-targeting therapeutic vaccines may be designed in a manner that effectively promotes the induction of clinically-relevant Type-1 antitumor CD8^+^ T cells in a manner that does not require the participation of CD4^+^ Th cells that are likely functionally sub-optimal or inappropriately skewed (e.g., induced Treg) in the tumor-bearing host.

Interleukin (IL)-15, a priority agent for cancer therapy,^5^ has been explored to improve the efficacy of vaccines, chemotherapies and adoptive T cell transfer approaches due to its ability to support DC, B cell, T cell and NK cell functionality, and to rescue tolerant or dysfunctional CD8^+^ T cells.^6-12^ Unfortunately, high-doses of *IL-15* (necessary for its bioactivity *in vivo*) via systemic administration of recombinant IL-15 protein (rIL-15) or overexpression of transgene *IL-15* have untoward side-effects [e.g., stimulating tumor cell growth, activating negative regulators (e.g., programmed death-1) in CD8^+^ T cells, exacerbating xenogeneic graft-vs.-host-disease or autoimmunity, and functioning as an “oncogene” resulting in progressive CD8^+^ T or NK leukemia],^13-17^ which have served to limit its benefit-to-risk ratio in the clinic, despite pre-clinical findings supporting the safety of rIL-15 in rhesus macaques.^18^ IL-15 agonists (e.g., IL-15/IL-15Rα-Fc complex and IL-15/IL-15Rα fusion protein) reduce the dose of delivered *IL-15* required to reach biologically-meaningful levels *in vivo*.^19-23^ However, cell (particularly DC) contact-dependent trans-presentation of membrane-bound IL-15/IL-15Rα appears required for optimal IL-15-mediated signaling *in vivo*.^24,25^

*In vivo*, *IL-15* derived from DC (DCIL-15) can “auto”-activate DC and substitute for the functional licensing events normally associated with DC interaction with CD4^+^ Th during vaccine activation of durable high-avidity CD8^+^ T cells, even though the mechanisms underlying this biology remain unknown.^10,26-30^ IL-15 is produced by cells (e.g., DC) at very low levels under normal physiologic conditions. The *in vivo* delivery of transgene *IL-15* into DC, which co-express full-length transgenic tumor Ag to allow for simultaneous DC presentation of Ag to T cells, may result in safer and more effective therapeutic vaccines that constitute an urgent, but as yet unmet, clinical need.

We have developed a novel DCIL-15-based cancer vaccine platform in which DC specifically express human *IL-15* transgene and simultaneously produce tumor Ag fused to human heat shock protein 70 (*Aghsp70*) as a specific immunogen, and demonstrated its potent antitumor effects in the prophylactic setting.^10^ In the current study, we found that DCIL-15 was superior to rIL-15 in improving both murine and human DC functions and potentiating therapeutic cancer vaccine efficacy in multiple clinically-relevant transplantable murine tumor models, and, notably, in the genetically engineered *Braf^V600E^*/*Pten*-driven melanoma murine model that recapitulates human disease.^31^ Results from these pre-clinical studies support the translational potential of this vaccine strategy for the future treatment of patients with cancer.

## Materials and Methods

### Mice, cell lines, and plasmids

C57BL/6 (B6)- or BALB/c-wild type (WT) and -Batf3^−/−^ and BALB/c-Foxp3-GFP mice (female, 6–8 weeks) were purchased from JAX (Bar Harbor, ME) or Taconic (Rensselaer, NY). B6-CD4^−/−^ mice (JAX) were backcrossed to the BALB/c for 12 additional generations. B6-Tyr-Cre^ERT2^Braf^CA^Pten^lox/lox^, -IL-15^−/−^, -IL-15Rα^−/−^, and -IL-2Rβ (CD122)^−/−^ mice were described previously.^31-33^ Mice were housed and bred in specific pathogen-free conditions in the University of Pittsburgh animal facility (Pittsburgh, PA). All animal procedures were performed according to approved protocols and in accordance with recommendations for the proper use and care of laboratory animals.

Murine melanoma B16 (ATCC, Manassas, VA) and glioma GL26^34^ cells were maintained in DMEM (IRVINE Scientific, Santa Ana, CA) supplemented with 10% fetal bovine serum (FBS) (Hyclone, Logan, UT), 2 mmol/l glutamine (Invitrogen, Carlsbad, CA) and 1×antibiotic antimycotic solution (Sigma, St Louis, MO). Murine breast tumor 4T1.2-Neu cells^35^ were cultured in the aforementioned medium including G-418 (500 μg/mL) (Invitrogen).

Plasmids expressing tumor-associated murine self Ag tyrosinase-related protein 2 (TRP2), rat oncoantigen Neu extracellular domain (NeuED) or human cancer-testis Ag MAGEA3 fused to human hsp70 [TRP2hsp70 (T7), NeuEDhsp70 (N7) or MAGEA3hsp70 (M7)] were described previously.^35-37^ DCIL-15-based DNA vaccines expressing human *IL-15*^38^ driven by DC-specific CD11c promoter (DCIL-15) and TRP2hsp70 or NeuEDhsp70 driven by constitutive CMV promoter [DCIL-15/TRP2hsp70 (DCIL-15/T7), DCIL-15/NeuEDhsp70 (DCIL-15/N7)] were constructed.^10^ All DNA were prepared using endotoxin-free DNA purification kit (Qiagen).

### DC generation and modification

*Mouse bone marrow (BM)-derived DC*: BM cells (2-3 × 10^6^/mL) from naive BALB/c-WT, and B6-WT, -IL-15^−/−^, -IL-15Rα^−/−^ or -IL-2Rβ^−/-^ mice were cultured in DC medium [RPMI1640 (IRVINE Scientific) supplemented with 10% FBS, 2 mmol/l glutamine, 1×antibiotic antimycotic solution, recombinant mouse granulocyte-macrophage colony stimulating factor (GM-CSF) (1,000 U/mL) and IL-4 (1,000 U/mL) (PeproTech)^11^]. On day 5–6, DC were purified using anti-mouse CD11c microbeads (Miltenyi Biotec, Auburn, CA). Purified DC (2–3 × 10^6^) were untreated or transfected with 7 μg endotoxin-free DNA using Amaxa mouse DC Nucleofector kit (Lonza) according to vendor's instructions. DNA-modified DC were continually cultured in 1 mL DC medium for 2 d before *in vitro* analyses or *in vivo* vaccinations. N7 or T7 DNA-modified DC were cultured in DC medium supplemented with 10 ng/mL rhIL-15 (R&D System) or clinical-grade rhIL-15 (NCI).

*Human monocyte-derived DC (moDC)*: Immature human moDC were generated from peripheral blood mononuclear cells obtained from adult healthy donors with written consent under an Institutional Review Board-approved protocol in DC medium [AIM V medium (invitrogen) supplemented with rhuIL-4 (20 ng/mL) (PeproTech) and clinical-grade rhuGM-CSF (Leukine®) (1000 U/mL) (Bayer)].^39^ On day 5–6, moDC (3 × 10^6^) were untreated or transfected with 7 μg endotoxin-free DNA using Amaxa Human DC Nucleofector kit (Lonza) according to vendor's instructions, and continually cultured in 1 mL DC medium for 2 d before *in vitro* analyses. M7 DNA-modified DC were cultured in DC medium supplemented with 10 ng/mL rhIL-15 or clinical-grade rhIL-15.

*Human skin-derived DC*: Human skins from surgical discard were obtained in accordance with the guidelines and the protocol approved by the Institutional Review Board of the University of Pittsburgh. Human skin epidermal/dermal explants were freshly prepared from skins with skin graft knife and subsequently untreated or immunized by 0.6 μm gold particles (BioRad) conjugated with IL-15/M7 or M7 DNA using a gene gun (GG) in sterile conditions.^40^ Clinical-grade rhIL-15 (10 ng in 10 μl AIM V medium) was intradermally (i.d.) injected into ∼4–5 cm^2^ M7-immunized human skin explants or M7-immunized human skin explants were cultured in AIM V medium supplemented with 10 ng/mL rhIL-15 or clinical-grade rhIL-15 (rIL-15/M7). After vaccination, these explants were cultured on sterile steel mesh with the epidermal side up in AIM V medium including 1×antibiotic/antimycotic solution at 37°C in 5%CO_2_. 3 d later, skin emigration cells were harvested from culture medium. Skin DC harvested from untreated or DNA-immunized human skin explants culture medium were stained by anti-HLA-DR-alexa flour 488, -CD14-brilliant violet 570, -IL-15-percep-cy5.5, and IL-15Rα-PE or isotype antibodies (BD Biosciences, eBioscience, Biolegend) and analyzed by flow cytometry on a BD LSRII. CD14^+^ DC were isolated from harvested skin DC using The EasySep™ Human CD14 Positive Selection Kit (Stem Cell Technologies).

### CD8^+^ T cell activation

*Naïve mouse CD8^+^ T cell activation*: Untreated or DNA-modified DC (1 × 10^5^) were cocultured with syngeneic CD8^+^ T cells (2.5 × 10^5^) isolated from the splenocytes of naïve BALB/c mice using anti-mouse CD8 microbeads (Miltenyi) in 200 μl RPMI1640 10%FBS in a 96-well plate in the presence or absence of Treg (GFP^+^) (2 × 10^5^) sorted from the spleen and tumor-draining lymph nodes (tdLN) of 4T1.2-Neu-bearing BALB/c-Foxp3-GFP mice as described previously.^41^ In some groups, functional anti-IL-15 and -IL-15Rα antibodies (5 μg/mL each) (R&D System) or goat IgG (Sigma) were added. 10 d later, murine IFNγ in the culture supernatants was determined by ELISA (BD Biosciences).

*Human CD8^+^ T cell activation*: Untreated or DNA-modified moDC (2 × 10^4^) were cocultured with autologous human CD8^+^ T cells (1 × 10^5^) isolated from lymphocytes using human CD8^+^ T cell isolation kit (Miltenyi) in 200 μl human T cell medium [IMDM supplemented with L-glutamine, penicillin, streptomycin, and nonessential amino acids (invitrogen) 10% human AB serum (Cellgro)]^39^ in the presence or absence of Treg (1 × 10^5^) isolated from autologous lymphocytes using human CD4^+^CD25^+^Treg isolation kit (Miltenyi) with two round purification.^42^ In some groups, functional anti-IL-15 and -IL-15Rα antibodies (5 μg/mL each) (R&D System) or goat IgG were added. On day 6 of DC-T cell coculture, T cells were restimulated with M7-modified autogolous moDC (2 × 10^4^) for other 6 d. Skin CD14^+^ DC or CD14^−^DC (1 × 10^5^) were cocultured with allogeneic human CD8^+^ T cells (5 × 10^5^) for 5 d. Human IFNγ in the culture supernatants was measured by ELISA (BD Biosciences).

### Treg function

*DC-modulated attenuation of suppressive activity of tumor-associated Treg:* Untreated or DNA-modified DC (1 × 10^5^) were cocultured with Treg (GFP^+^) (2 × 10^5^) sorted from the spleen and tdLN of 4T1.2-Neu-bearing BALB/c-Foxp3-GFP mice.^41^ 2 d later, Treg were isolated by anti-mouse CD4 microbeads (Miltenyi) from pooled DC-Treg coculture. The ability of Treg to suppress T cell activation *in vitro* was measured as described previously^41^: 4T1.2-Neu-primed CD4^+^ T cells (2 × 10^5^), 4T1.2-Neu lysate-loaded naïve BALB/c splenic DC (2 × 10^5^) and naïve BALB/c splenic CD8^+^ T cells (2 × 10^5^) were cocultured with or without Treg (2 × 10^5^) for 5 d.

*Braf^V600E^/Pten-driven melanoma-induced activation of Treg*: *Braf^V600E^/Pten*-driven melanoma was developed by inducing oncogene *Braf^V600E^* expression with 4-hydroxytamoxifen (4-HT) (H6278, Sigma) in *B6-Tyr-Cre^ERT2^Braf^CA^Pten^lox/lox^* mice with correct genotype (presence of *Tyr-Cre^ERT^*^2^, *Braf^CA^*, and homozygous *Pten^lox/lox^*).^31^ Treg were purified from single-cell suspensions of tdLN of *Braf^V600E^/Pten*-driven melanoma-bearing mice (tdLN Treg) using mouse Treg isolation kit (Miltenyi). Intratumoral Treg were purified from tumor-infiltrating lymphocytes (TIL), isolated from pooled single-cell suspensions of melanoma obtained by digesting with collagenase D (Roche) (1 mg/mL in RPMI 1640) with a standard Ficoll® density separation, using mouse Treg isolation kit. The ability of these Treg to *in vitro* suppress T cell activation was determined as described previously ^41^: melanoma-primed CD4^+^CD25^−^T cells (2 × 10^5^) from tdLN, melanoma lysate-loaded naïve B6 splenic DC (2 × 10^5^) and naïve B6 splenic CD8^+^T cells (2 × 10^5^) were cocultured with or without tdLN Treg (2 × 10^5^) or intratumoral Treg (1 × 10^4^) for 5 d. **Murine IFNγ in the culture supernatants was measured by ELISA**.

### Therapeutic melanoma (TRP2)-specific CD8^+^ T cell responses

Braf^V600E^/Pten-driven melanoma (∼3 mm)-bearing B6-Tyr-Cre^ERT2^Braf^CA^Pten^lox/lox^ mice (2–3/group) were untreated or vaccinated using a GG with DCIL-15/T7 or T7 DNA on days 0, 7 and 14 as described previously.^10,43^ T7 DNA-vaccinated mice were intraperitoneally (i.p.) daily injected with clinical-grade rhIL-15 (NCI) [2.95 μg in 100 μl endotoxin-free 1×PBS (Sigma)/injection] for 3 d post each vaccination (rIL-15/T7). On day 60, single cell suspensions of tdLN were stained with anti-CD8-pacific blue, -CD44-FITC and -CD62L-PE or isotype control antibody (eBioscience, BD Biosciences) and analyzed by flow cytometry. At the same time, CD8^+^T cells were purified from splenocytes and tdLN using anti-mouse CD8 microbeads. Purified CD8^+^ T cells (2 × 10^5^) were cocultured with syngeneic BM DC (4 × 10^4^) transfected by T7 DNA or pulsed by *Braf^V600E^/Pten*-driven melanoma lysates (N7-transfected and 4T1.2-Neu lysate-pulsed DC as controls) in 200 μl RPMI 1640 10% FBS at 37°C, 5% CO_2_ for 3 d. Murine IFNγ in the culture supernatants was determined by ELISA.

### Therapeutic vaccinations

#### Transplantable melanoma B16, glioma GL-26, and breast carcinoma 4T1.2-Neu models

*DC vaccines*: BALB/c-WT or -CD4^−/−^ or B6 mice (3/group) were subcutaneously (s.c.) inoculated with exponentially growing 4T1.2-Neu (2 × 10^4^) at the 4th mammary fat pad or GL26 (1 × 10^6^) at right flank on day 0.^10,43^ On day 8, tumor-bearing mice were randomly allocated to be untreated or i.p. immunized once by the various vaccine DC (2.5 × 10^5^).

*DNA vaccines*: B6- or BALB/c-WT, -Batf3^−/−^ mice (3–5/group) were inoculated s.c. with B16 (4×10^4^), GL26 (1 × 10^6^), or 4T1.2-Neu (2 × 10^4^) on day 0. On day 8 or 9, tumor-bearing mice were randomly allocated to be untreated or vaccinated by a GG with DNA once or 2–3 times weekly (details in figure legends).^10,43^ To deplete CD8^+^ T cells, anti-mouse CD8 mAb (53–6.7) (200 μg/injection) were i.p. injected on days 6, 9, 14 and 21. Plasmacytoid DC (pDC) were depleted by i.p. injection of 200 μg anti-mouse pDC Ab (120G8) (BioX Cell) 1 day before, on the day of, and 1 day after vaccination.

*Genetically engineered Braf^V600E^/Pten-driven melanoma model* *Braf^V600E^/Pten*-driven melanoma, which was developed by inducing oncogene *Braf^V600E^* expression with *4-HT* in *B6-Tyr-Cre^ERT2^Braf^CA^Pten^lox/lox^* mice with correct genotype,^31^ was allowed to grow progressively to a mean tumor size of ∼3 mm, at which time, melanoma-bearing mice were randomized into cohorts of 3–4 mice with each cohort exhibiting a comparable mean tumor size. Mice were then left untreated or they were vaccinated using a GG with DCIL-15/T7 or T7 DNA on days 0, 7 and 14.^10,43^ Melanoma-bearing mice immunized by T7 DNA were i.p. injected with clinical-grade rhIL-15 for 3 d immediately after each immunization as described above. Endogenous CD8^+^ T cells were depleted by i.p. injection of anti-mouse CD8 mAb 1 day before, on the day of, and 1, 3 d after first vaccination, and then weekly.

In all therapeutic experiments, tumors were measured every 3 d using a digital slide calipers (Fisher Scientific, Pittsburgh, PA) in the two perpendicular diameters. Mice were followed until their unanticipated (natural) death or they were euthanized when tumor reached a mean size of 10 mm. On day 30 after 4T1.2-Neu inoculation, mice were sacrificed and lungs were fixed with Bouin's solution (Sigma) for counting tumor foci. At the same time, tumors in BM were selected *in vitro* with 4T1.2-Neu culture medium as described above.^35^

### Statistical analysis

Data were statistically analyzed using Student's *t*-test (immune assays, tumor size and loci) (Graph Pad Prism version 6). Data from animal survival experiments were statistically analyzed using Log rank test (Graph Pad Prism version 6). Animal survival is shown by Kaplan-Meier Survival Curves. *P* < 0.05 is considered to be statistically significant. *p <0.05; **p <0.01; ***p <0.001; N.D. (not detected).

## Results

### DCIL-15-based DNA vaccines elicit potent CD8^+^ T cell-dependent therapeutic antitumor immunity in multiple clinically-relevant murine tumor models

**Figure 1.**
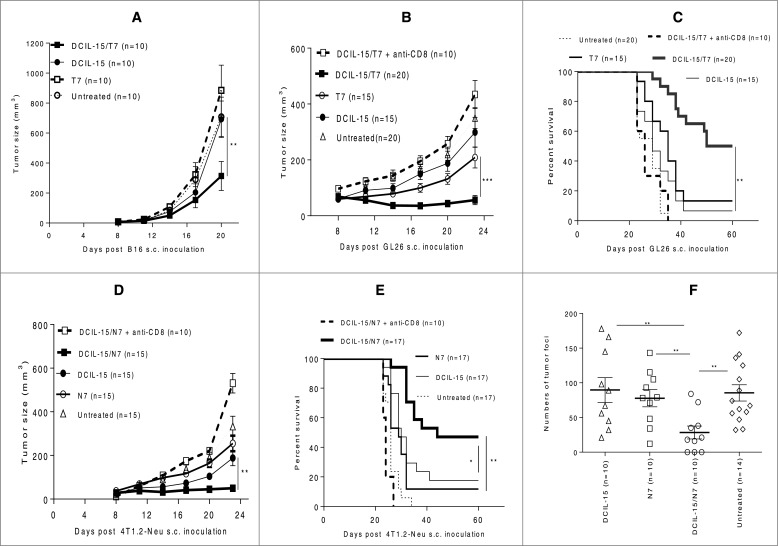
DCIL-15-based DNA vaccines elicit CD8^+^ T cell-dependent therapeutic antitumor immunity against primary tumors and lung metastases. B6 and BALB/c mice were inoculated with B16 (**A**) and GL26 (**B and C**) or 4T1.2-Neu (**D and E**) on day 0. Tumor-bearing mice were randomly allocated to be untreated or vaccinated by a GG with DNA twice on days 8 and 15 (**A**) or 3 times on days 8, 15 and 22 (**B–E**). To deplete endogenous CD8^+^ T cells, anti-mouse CD8 mAb were injected on days 6, 9, 14 and 21 (**B–E**). BALB/c mice were s.c. injected with 4T1.2-Neu on day 0 and untreated or vaccinated by a GG with DNA once on day 8, and tumor foci in lungs were counted on day 30 (**F**). Data from three (**A and D**), four (**B, C, and E**), or two (**F**) independent experiments are shown and were statistically analyzed.

We have designed a novel DCIL-15-based cancer vaccine platform in which DC specifically express human *IL-15* transgene and simultaneously produce tumor *Aghsp70*, and demonstrated its efficacy in effectively inducing prophylactic antitumor immunity.^10^ When incorporating the tumor-associated Ag TRP2 or NeuED, we found that DCIL-15-based combination DNA vaccines elicited potent therapeutic antitumor immunity against distinct murine established tumors (i.e., 8 d after s.c. inoculation of a lethal-dose of the various tumor cells) including syngeneic native melanoma (B16) (**Fig. 1A***)*, glioma (GL26, naturally expressing TRP2) ^34^ (**Fig. 1B and C**), and spontaneous metastatic breast tumor (4T1.2-Neu, 4T1.2 ectopically expressing activated onco-antigen rat Neu) ^35^ (**Fig. 1D–F**, **Table 1**). Furthermore, antibody-based CD8^+^ T cell depletion abrogated the protective effects associated with vaccination (**Fig. 1B–E**). These results suggest the broad therapeutic potency of this vaccine strategy in eliciting host protective CD8^+^ T cell responses against primary and metastatic tumors.

**Table 1.** A single immunization with DCIL-15-based DNA vaccine inhibits tumor cell BM metastases

DNA vaccines	Mice with BM metastases/mice used in experiments (%)	
DCIL-15	7/10 (70%)	4T1.2-Neu-bearing BALB/c mice were untreated or immunized (**Fig. 1F**). Day 30, 4T1.2-Neu tumor cells in BM were selected with 4T1.2-Neu culture medium ^35^. Data combined from two independent experiments are shown and were statistically analyzed (Fisher's exact test, Graph-Pad InStat). DCIL-15/N7 vs. untreated, N7, or DCIL-15: *p* < 0.05.
N7	6/10 (60%)	
DCIL-15/N7	2/10 (20%)	
Untreated	11/14 (78%)

### Therapeutic antitumor immunity elicited by DCIL-15-based DNA vaccines depends on host Batf3^+^ DC subsets and partially requires the pDC subset of antigen-presenting cells

**Figure 2.**
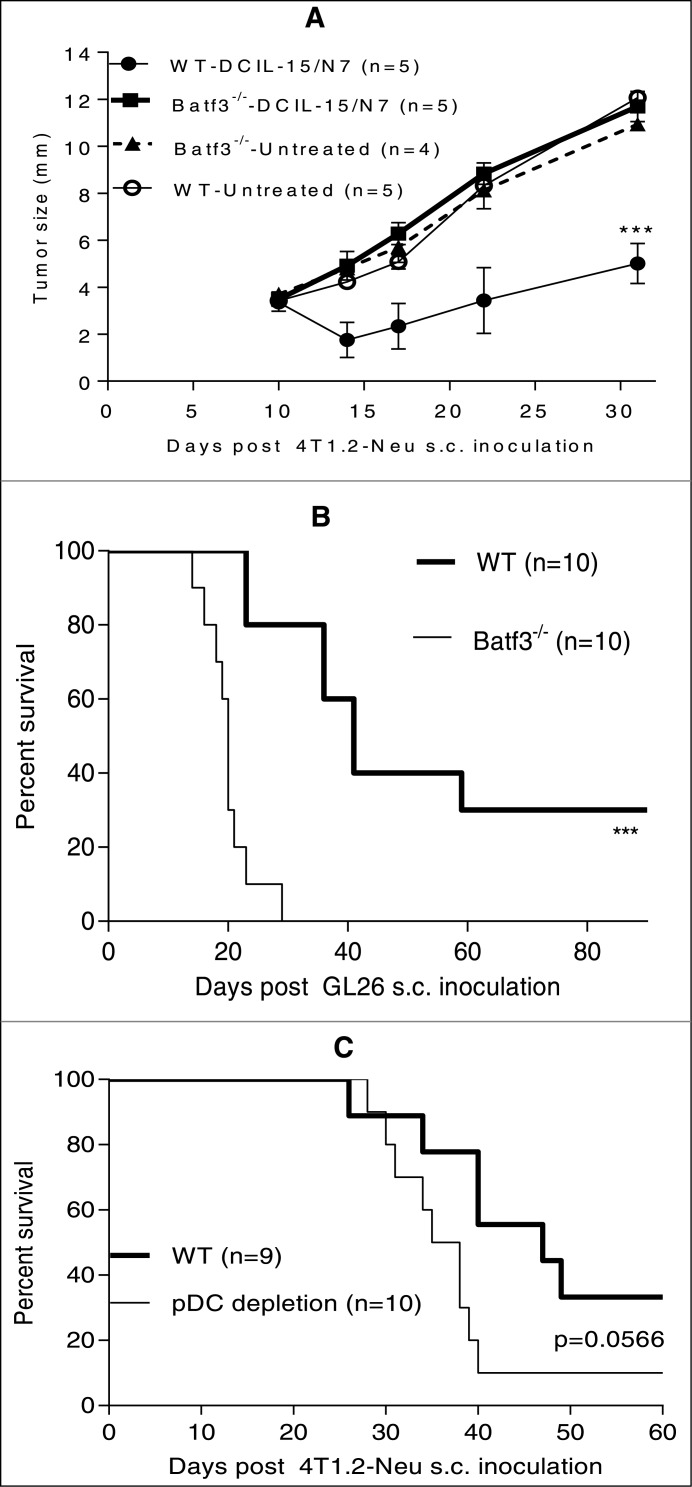
Therapeutic antitumor immunity elicited by DCIL-15-based DNA vaccines depends on Batf3^+^ DC and partially requires pDC. BALB/c and B6-WT and -Batf3^−/−^ mice were inoculated with 4T1.2-Neu (**A and C**) or GL26 (**B**) on day 0. Tumor-bearing mice were randomly allocated to be untreated or vaccinated by a GG with DNA once on day 8 (**A and B**) or twice on days 7 and 14 (**C**). pDC were depleted by injection of anti-mouse pDC Ab 1 d before, on the day of, and 1 day after each vaccination (**C**). Data from two independent experiments are shown and statistically analyzed.

Batf3^−/−^ mice are selectively deficient of the antigen cross-presenting CD8α^+^ and CD103^+^DC subsets,^44^ which are required for *in vivo* priming of tumor-specific CD8^+^ T cells.^45,46^ Using these mice as tumor-bearing hosts, we observed that therapeutic antitumor immunity elicited by DCIL-15-based DNA vaccines required host Batf3^+^ DC subsets in both the GL26 and 4T1.2-Neu models (**Fig. 2A and B**). Since *IL-15* may mediate cross-talk between pDC and conventional DC (cDC) during the course of specific T cell activation,^47^ we next investigated the requirement for pDC in vaccine efficacy. Depletion of pDC during the immunization protocol partially impaired the ability of therapeutic intervention to promote antitumor immunity (**Fig. 2C**). These data indicate that the optimal therapeutic antitumor immune response promoted by this immunization approach requires host Batf3^+^ DC and the participation of pDC.

**DCIL-15 (not rIL-15)-based DNA vaccines elicit robust durable therapeutic CD8^+^ T cell responses in a clinically-reflective *Braf^V600E^/Pten*-driven melanoma model**.

DCIL-15-based DNA vaccines were therapeutically effective in transplantable murine B16 melanoma, GL26 glioma, and 4T1.2-Neu breast carcinoma models (**Fig. 1**, **Table 1**). Given the contention that findings from transplantable tumor models may not provide the most useful information for translation into the clinic, we next evaluated a genetically engineered inducible *Braf^V600E^/Pten*-driven melanoma murine model that is more reflective of human disease (e.g., tumor self Ag expression, highly metastatic, and relapses post chemotherapies).^31^ Melanoma in this model develops when oncogenic *Braf^V600E^* expression in melanocytes is induced by treatment with *4-HT*.^31^ As observed in many aggressive solid tumors, Foxp3^+^CD4^+^Treg infiltrated into *Braf^V600E^/Pten*-driven melanoma and melanoma-associated tdLN and intratumoral Treg exhibited potent activity of suppressing T cell activation (**Fig. S1**).

**Figure 3.**
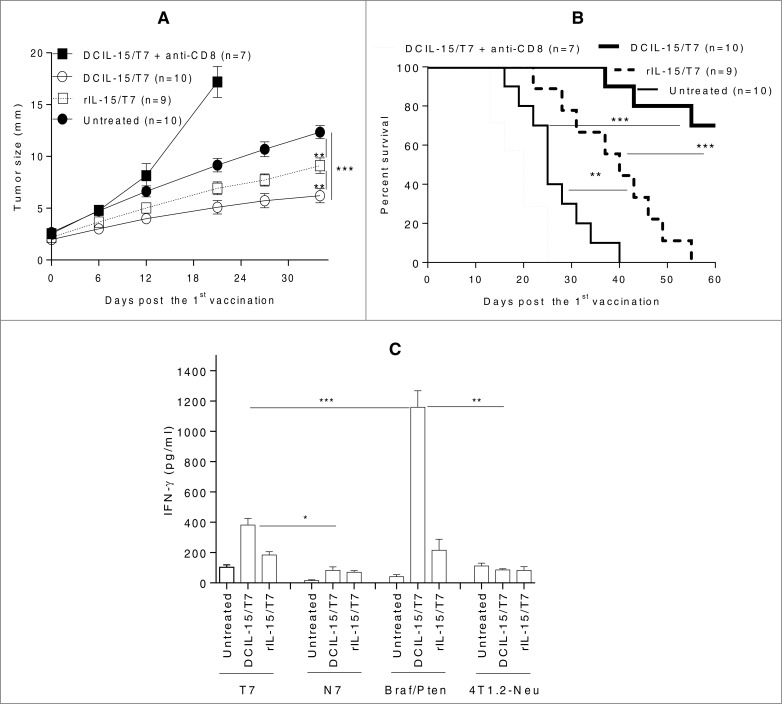
DCIL-15 (not rIL-15)-based DNA vaccines induce durable therapeutic CD8^+^ T cell-dependent antitumor immunity in a *Braf^V600E^/Pten*-driven melanoma model. *Braf^V600E^/Pten*-driven melanoma (∼3 mm)-bearing *B6-Tyr-Cre^ERT2^Braf^CA^Pten^lox/lox^* mice were untreated or vaccinated by a GG with DCIL-15/T7 or T7 DNA on days 0 (1st immunization), 7 and 14. Melanoma-bearing mice immunized by T7 DNA were injected with clinical-grade rhIL-15 for 3 d post each vaccination. Endogenous CD8^+^ T cells were depleted by injection of anti-mouse CD8 mAb 1 day before, on the day of, and 1, 3 d after first vaccination, and then weekly. Tumor growth (**A**) and animal survival (**B**) were monitored. On day 60, CD8^+^ T cells purified from splenocytes and tdLN were cocultured with syngeneic BM DC modified by T7 DNA or pulsed by *Braf/Pten* melanoma lysates (N7 DNA-modified and 4T1.2-Neu lysate-pulsed syngeneic DC as controls) (**C**). IFNγ in the culture supernatants was determined by ELISA. Data from three independent experiments are shown and were statistically analyzed.

Either DCIL-15- or rIL-15-based DNA-based vaccines generated effective therapeutic antitumor immunity against established transplantable GL26 glioma (**Fig. S2**). Indeed, rIL-15-based DNA vaccines were slightly superior to DCIL-15-based DNA vaccines in this model (**Fig. S2**). In contrast, in the established transgenic *Braf^V600E^/Pten*-driven melanoma model, DCIL-15-based DNA vaccines were significantly more effective than rIL-15-based DNA vaccines in controlling tumor growth, and, notably, prolonging the survival of tumor-bearing mice (**Fig. 3A and B**). CD8^+^ T cell depletion markedly abrogated vaccine-induced therapeutic immunity (**Fig. 3A and B**). Accordingly, DCIL-15 (not rIL-15)-based DNA vaccines elicited durable melanoma (TRP2)-specific IFNγ-producing CD8^+^ T cell responses (**Fig. 3C**). These results indicate that DCIL-15 is superior to rIL-15 in potentiating the antitumor efficacy of DNA-based cancer vaccines, leading to CD8^+^ T cell-dependent antitumor immunity against clinically-reflective *Braf^V600E^/Pten*-driven melanomas.

### DC genetically-modified to express *IL-15* and *Aghsp70* are superior to the DC genetically-modified to express only *Aghsp70* and cultured with rIL-15 in activating CD8^+^ T cells

**Figure 4.**
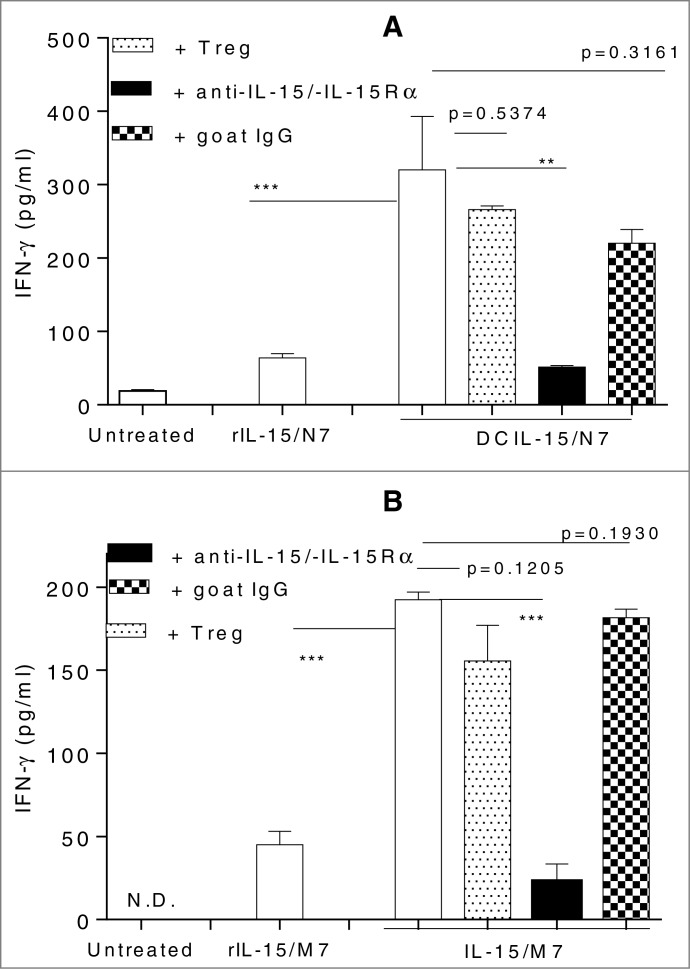
DC genetically-modified to express both *IL-15* and *Aghsp70* are superior to the DC genetically-modified to express only *Aghsp70* and cultured with rIL-15 in activating autologous CD8^+^ T cells, and function in a CD4^+^ Th-independent, but IL-15/IL-15Rα-dependent manner, even in the face of Treg-mediated suppression. Mouse BM DC were untreated or modified with DCIL-15/N7 or N7 DNA. N7-modified DC were cultured with rhIL-15 (rIL-15/N7). After 2 d, those DC were cocultured with syngeneic naïve CD8^+^ T cells in the presence or absence of tumor-associated Treg, functional anti-*IL-15* and -IL-15Rα Abs, or goat IgG for 10 d (**A**). Human moDC were untreated or modified with IL-15 and M7 (IL-15/M7) or M7 DNA. M7-modified DC were cultured with rhIL-15 (rIL-15/M7). After 2 days, DC were cocultured with autologous human CD8^+^ T cells in the presence or absence of activated autologous Treg, functional anti-*IL-15* and -IL-15Rα Abs, or goat IgG. On day 6 of the coculture, M7-modified autologous moDC were added for restimulation of other 6 d (**B**). IFNγ in the culture supernatants was determined by ELISA. Data from two independent experiments are shown and were statistically analyzed.

*IL-15* gene therapy and rIL-15 are being used to generate *ex vivo* DC vaccines for cancer immunotherapy in preclinical models and clinical trials/studies.^8,10,11^ We have previously shown that murine BM DC genetically-modified to express *IL-15* and *Aghsp70* secrete substantial levels of human *IL-15* and produce *Aghsp70*, in association with their enhanced maturation as evidenced by higher levels of cell surface IL-15Rα expression.^10^ Although both murine BM DC and human moDC genetically-engineered by *Aghsp70* (i.e., N7 for murine DC, M7 for human DC) and cultured with rIL-15 (without other maturation factors) (rIL-15-based DC) were able to activate syngeneic/autologous CD8^+^ T cells without the need for CD4^+^ Th “help,” DC genetically-modified to express both *IL-15* and *Aghsp70* (DCIL-15-based DC) were much more effective in activating cognate syngeneic CD8^+^ T cells (**Fig. 4**). Neutralization of IL-15/IL-15Rα signal using specific anti-IL-15 and -IL-15Rα antibodies during the *in vitro* DC-CD8^+^ T cell coculture abolished DC-induced activation of CD8^+^ T cells (**Fig. 4**), and such activation circumvented the suppressive effects of Treg (**Fig. 4**). These results suggest that DCIL-15 is superior to rIL-15 in activating DC for the activation of CD8^+^ T cells without a need for CD4^+^ Th “help,” but in an IL-15/IL-15Rα-dependent manner that overcomes suppression mediated by Treg.

### DCIL-15 (not rIL-15)-based DC attenuate tumor-associated Treg and induce therapeutic antitumor immunity in an IL-15Rα- or IL-2Rβ-dependent manner that does not require endogenous production of IL-15 or CD4^+^ T cell “help”

**Figure 5.**
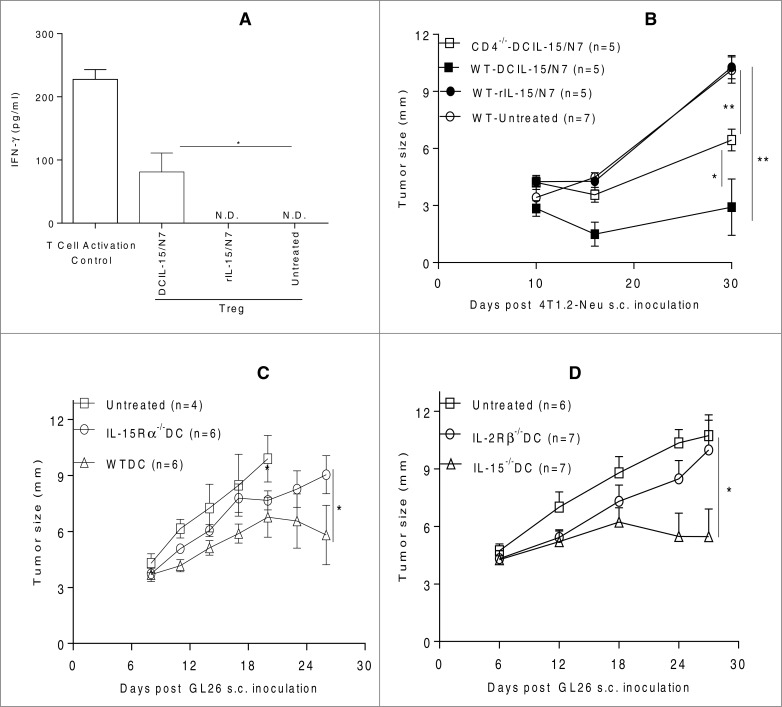
DC genetically-modified by *IL-15*/*Aghsp70* attenuate tumor-associated Treg and induce therapeutic antitumor immunity in an IL-15Rα or IL-2Rβ-dependent but do not require endogenous IIL-15 production or CD4^+^ T cell “help.” Mouse BM DC were untreated or modified with DCIL-15/N7 or N7 DNA. N7-modified DC were cultured with rhIL-15 (rIL-15/N7). After 2 d, those DC were cocultured with tumor-associated Treg for 2 d and then the ability of those DC-modulated Treg to suppress T cell activation was determined (**A**). BALB/c-WT or -CD4^−/−^ (**B**) and B6 (**C and D**) mice were inoculated with 4T1.2-Neu (**B**) or GL26 (**C and D**). On day 8, tumor-bearing mice were randomly allocated to be untreated or immunized once by DCIL-15/N7 or rIL-15/N7 DC (**B**) or DC-WT, -IL-15^−/−^, -IL-15Rα^−/−^ or -IL-2Rβ^−/−^ modified by DCIL-15/T7 (**C and D**). Tumor size was monitored. Data from two independent experiments are shown and were statistically analyzed.

Without the addition of alternate DC maturation signals, DCIL-15 (but not rIL-15)-based DC were observed to attenuate the suppressive activity of tumor-associated Treg *in vitro* (**Fig. 5A**). *In vivo*, a single low-dose of the DCIL-15 (not rIL-15)-based DC vaccines generated therapeutic antitumor immunity even in the CD4^+^ Th-deficient tumor-bearing hosts (**Fig. 5B**). Therapeutic antitumor immunity induced by DCIL-15-based DC vaccines was significantly associated with expression of IL-15Rα or IL-2Rβ on vaccine DC (**Fig. 5C and D**). Furthermore, DCIL-15-modified IL-15^−/-^ DC were capable of generating effective therapeutic antitumor effects (**Fig. 5D**).

**CD14^+^ DC emigrating from human skin explants genetically-immunized by *IL-15* and M7 are more effective than the DC emigrating from the explants genetically-immunized by M7 alone in the presence of rIL-15 in expressing membrane-bound IL-15/IL-15Rα and activating CD8^+^ T cells**.

**Figure 6.**
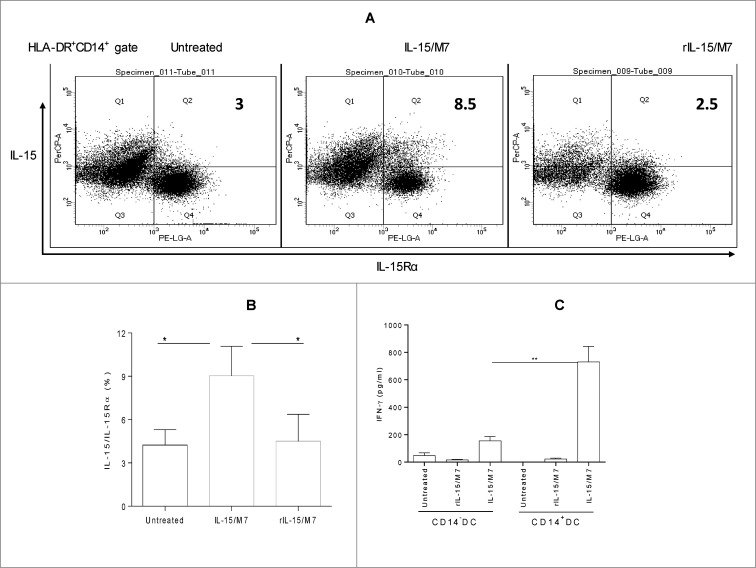
CD14^+^ DC emigrating from human skin explants genetically-immunized by *IL-15* and M7 are more effective than the DC emigrating from the explants genetically-immunized by M7 in the presence of rIL-15 in enhancing IL-15/IL-15Rα expression and CD8^+^ T cell activation. Skin emigration cells harvested from culture medium of human skin explants untreated or immunized by IL-15/M7 or M7 DNA in the presence of rhIL-15 (rIL-15/M7) were stained by anti-HLA-DR, -CD14, -IL-15, and -IL-15Rα and analyzed by flow cytometry (**A and B**). CD14^+^ DC or CD14^−^ DC isolated from skin emigration cells were cocultured with allogeneic human CD8^+^ T cells for 5 d, and IFNγ in the culture supernatants was measured by ELISA (**C**). One representative (IL-15/IL-15Rα expressing on HLA-DR^+^CD14^+^ cells) of three independent experiments with a similar result is presented (**A**). Data from three (**B and C**) independent experiments are shown and were statistically analyzed.

Skin harbors multiple DC subsets and is an ideal anatomic site for vaccination of Type-1 immune responses. DC that leave human skin explants *in vitro* are believed to be analogous to tissue DC that travel to LN *in vivo* after locoregional antigenic insult. CD14^+^ DC (not CD14^−^ DC) emigrating from human skin explants genetically-immunized by *IL-15* and M7 enhanced membrane-bound IL-15/IL-15Rα expression when compared to the explants untreated or genetically-immunized by M7 in the presence of rIL-15 (**Fig. 6A and B**, data not shown). Although both CD14^+^ DC and CD14^−^ DC emigrating from human skin explants genetically-immunized by *IL-15* and M7 were more effective than the DC emigrating from the explants genetically-immunized by M7 alone in the presence of rIL-15 in activating allogeneic CD8^+^ T cells (**Fig. 6C**), CD14^+^ DC were superior to CD14^−^ DC in such activation. These results suggest that this vaccination strategy may be useful in potentiating the ability of human skin DC to drive therapeutic CD8^+^ T cell responses in the cancer setting.

## Discussion

*IL-15*, one of the most promising biological agents for cancer treatment,^6^ has been used in the *in vitro* culture (via r*IL-15*) or modification (via transgene *IL-15*) of CD8^+^ T cells, DC or tumor cells for generation of tumor-specific CD8^+^ T cells and DC- or tumor cell-based vaccines, and administered systemically *in vivo* by injection of rIL-15 or expression of transgene *IL-15* alone or in combination with other agents/approaches (e.g., IL-12, IL-21, IL-7, GM-CSF, anti-CD40, anti-PD-L1, anti-CTLA-4, adoptive CD8^+^T cell transfer, irradiation).^6-12,48-52^ The therapeutic antitumor efficacy of these *IL-15*-based strategies has been demonstrated in transplantable tumor models. The phase I/II clinical trials with rIL-15 are currently being performed in patients with various forms of cancer.^52^

The unique *in vivo* mechanism of action of *IL-15* occurs through cell, notably, DC contact-dependent trans-presentation of a membrane-bound IL-15/IL-15Rα signal to effector cells (e.g., NK and CD8^+^ T cells expressing IL-15Rβ/γ receptor) that is required for optimal IL-15-mediated signaling under physiologic conditions.^6,24,25,53^ Accordingly, *ex vivo*-generated DC or tumor cells co-expressing transgene IL-15 and IL-15Rα, and engineered IL-15 agonists (e.g., IL-15/IL-15Rα-Fc complex, IL-15/IL-15Rα fusion protein) have been explored to generate effective antitumor responses.^14,19-23,51^

DCIL-15 activates DC and provides signals equating to those normally provided by CD4^+^ Th in “licensing” DC for CD8^+^ T cell activation even though the underlying mechanisms associated with this biology remain largely unknown.^10,26-30^ Directed *in vivo* delivery of transgene *IL-15* into vaccine DC has the potential to enhance the potency of DCIL-15 provided in a trans-presented manner from vaccine DC to responder CD8^+^ T cells. Regulated provision of transgene *IL-15* in this manner may also obviate, or at least reduce, potential adverse events associated with nonspecific overexpression of transgene *IL-15 in vivo*.^14-17^

We observed that DCIL-15 was superior to rIL-15 in promoting both murine and human DC functionality and therapeutic cancer vaccine efficacy in multiple distinct and clinically-relevant murine tumor models (^10^, **Figs. 1****–****6**), suggesting the broad therapeutic potency of this vaccine strategy in eliciting antitumor immunity against primary tumors and their metastases.

DC-derived intracellular *IL-15* interacting with IL-15Rα (in *cis*) during production within DC results in mutual stabilization and increased bioactivity of membrane-bound IL-15/IL-15Rα on DC that may promote IL-15 action on IL-2Rβ (another receptor of IL-15) expressed by CD8^+^ T cells, leading to efficient IL-15/IL-15Rα *trans* signaling in support of enhanced antitumor CD8^+^ T cell activation. The lack of response to IL-15 stimulation by IL-2Rβ^−/−^-DC suggests that IL-2Rβ expressed by DC may play an important role in IL-15-mediated signaling.^54^ IL-15Rα substantially increases the affinity of IL-15 for IL-2Rβ, and this allosteric interaction is required for effective IL-15-mediated signaling into T cells.^55^ Thus the strategic enhanced production of transgene *IL-15* by IL-15Rα^+^ DC likely plays a major role in the superior ability of DCIL-15-based cancer vaccines to promote robust antitumor CD8^+^ T cell responses including those from central memory CD8^+^ T cells (CD8^+^CD44^hi^CD62L^hi^) and effector memory CD8^+^ T cells (CD8^+^CD44^hi^CD62L^lo^) in tdLN (**Fig. S3**). DC modified by DCIL-15/*Aghsp70* produced substantial levels of pro-inflammatory cytokines (e.g., IL-6),^10^ which may also assist in the inactivation of Treg.^56^

CD4^+^ Th are generally considered indispensable during the induction of optimal CD8^+^ T cell responses, however, our analyses of DCIL-15-based vaccines indicated that CD4 depletion did not impair vaccine efficacy in either the prophylactic^10^ or therapeutic settings (**Fig. 5C**). DCIL-15-based vaccines induced durable Type-1 (i.e. IFNγ-producing) CD8^+^ T cells reactive against the melanoma-associated Ag TRP2, even in CD4^+^ T cell-deficient mice (**Fig. S4**). The ability of this vaccine to work in the absence of CD4^+^ T “helper” cells may be translationally important in the context of cancer patients (e.g., HIV-infected and some after chemotherapy) who are frequently deficient in (Type-1) CD4^+^ Th function.^57,58^

CD14^+^ DC emigrating from human skin explants genetically-immunized by *IL-15* and *Aghsp70* enhanced their cell surface expression of IL-15/IL-15Rα and readily activated CD8^+^ T cells. Although it remains to be determined whether HLA-DR^+^CD14^+^IL-15^+^/IL-15Rα^+^ DC that emigrate out of human skin explants after genetic-immunization using this vaccine strategy promote the differentiation of melanoma Ag-specific CD8^+^ T cells *in vitro* and in humanized (human melanoma-bearing severe combined immunodeficiency) mice, our current data support the notion that this approach could be used to potentiate human skin DC for the effective genetic immunization against cancer.

Furthermore, although the current study focused on the influences of DCIL-15-based cancer vaccines on CD8^+^ T cells, NK cells are also responsive to IL-15, and the impact of DCIL-15-based cancer vaccines on Type-1 NK cells needs to be comprehensively evaluated in future studies.

Although the precise mechanisms behind the DCIL-15-based vaccine strategy need to better delineated, its merits are evident: a) Directed *in vivo* delivery of transgene *IL-15* into vaccine IL-15Rα^+^DC focuses DCIL-15 on activating DC, substituting for CD4^+^ Th, and potentiating vaccine efficacy. This may lead to the most efficient utilizing the cytokine IL-15 *in vivo* while minimizing concerns for off-target toxicities associated with systemic administration of rIL-15 or overexpression of transgene *IL-15*. b) The vaccines were therapeutically effective even in the CD4^+^ Th-deficient mice and this induction strategy circumvented Treg-mediated suppression. This may be important to the design of vaccines for cancer patients who are deficiency of CD4^+^ Th and/or display profound immune suppression. c) The possibility of a single-immunization of either small numbers of *ex vivo*-generated DCIL-15-based DC or *in vivo* DC-targeting DNA vaccines required to induce effective antitumor immunity avoids or at least minimizes the need for secondary immunizations that makes this vaccine strategy both feasible and translationally-attractive from a logistics perspective. d) The *in vivo* mouse data from multiple highly clinically-relevant tumor models including the authentic *Braf^V600E^/Pten*-driven melanoma and the *in vitro* human data from both blood- and skin-derived DC support the translational potential of this vaccine strategy in the clinic.

Given the current priority status for the clinical use of rIL-15 in patients with cancer and our findings for the superior performance of DCIL-15 over rIL-15 in cancer vaccine formulations, and DNA vaccines offering the potential for an off-the-shelf, easily-scalable vaccine platform, this vaccine strategy is both salient and attractive for translation as a future cancer therapeutic modality that could benefits the vast majority of cancer patients. This is particularly compelling for those patients with profound defects in CD4^+^ Th “helper” responses (or robust suppressor cell populations).
